# The design of an inkjet drive waveform using machine learning

**DOI:** 10.1038/s41598-022-08784-y

**Published:** 2022-03-22

**Authors:** Seongju Kim, Minsu Cho, Sungjune Jung

**Affiliations:** 1 Department of Mechanical Engineering, Pohang University of Science and Technology (POSTECH), 77 Cheongam-Ro, Nam-Gu, Pohang, 37673 Republic of Korea; 2 Department of Computer Science and Engineering, Pohang University of Science and Technology (POSTECH), 77 Cheongam-Ro, Nam-Gu, Pohang, 37673 Republic of Korea; 3 Department of Materials Science and Engineering, Pohang University of Science and Technology (POSTECH), 77 Cheongam-Ro, Nam-Gu, Pohang, 37673 Republic of Korea

**Keywords:** Mechanical engineering, Materials science

## Abstract

A drive waveform, which needs to be optimized with ink’s fluid properties, is critical to reliable inkjet printing. A generally adopted rule of thumb for its design is mostly dependent on time-consuming and repetitive manual manipulation of its parameters. This work presents a closed-loop machine learning approach to designing an optimal drive waveform for satellite-free inkjet printing at a target velocity. Each of the representative 11 model inks with different fluid properties was ink-jetted with 1100 distinct waveform designs. The high-speed images of their jetting behaviors were acquired and the big datasets of the resulting drop formation and velocity were extracted from the jetting images. Five machine learning models were examined and compared to predict the characteristics of jetting behavior. Among a variety of machine learning models, Multi-layer Perceptron affords the highest prediction accuracy. A closed-loop prediction algorithm that determined the optimal set of waveform parameters for satellite-free drop formation at a target velocity and employed the most superior learning model was established. The proposed method was confirmed through the printing of an unknown model ink with a recommended waveform.

## Introduction

Drop-on-demand inkjet printing has been widely embraced as a versatile production technology for both small and large formats of graphical and text printing^1^. As a maskless, non-contact patterning method, this additive manufacturing technology is increasingly being considered a crucial technology for new applications, such as next-generation sensors, circuits, displays, and biological tissues^2–6^. Unlike other printing processes that produce printed patterns by the transfer of ink from pre-defined master patterns^7–9^, inkjet printing builds up patterns directly on a substrate by depositing tiny drops of functional inks that contain a wide range of functional components such as metal nanoparticles, conducting polymers, or biological materials. A digitally controlled nozzle produces a series of drops with a diameter of typically 10–100 μm. As the name implies, a piezoelectric inkjet printhead uses a piezoelectric actuator to convert applied electrical energy into mechanical deformation of an ink chamber^10^. The displacement of the chamber wall controlled by a drive waveform produces the pressure required for a drop to form and eject from the nozzle. Many of the inkjet printing applications necessitate a reliable jetting behavior of functional ink, which is mainly determined by the ink fluid characteristics and drive waveform design^11–14^.

The ink-jetting process of fluids involves a variety of free surface deformation. Inertia, capillary force, and viscous dissipation are generally used to govern the generation of inkjet drops^15^. These phenomena can be characterized by dimensionless parametric groups such as Reynolds number (*Re*), Weber number (*We*), and Bond number (*Bo*)

1 $$Re = \frac{Inertial\,force}{{Viscous\,force}} = \frac{\rho vD}{\eta }$$

2 $$We = \frac{Inertial\,force}{{Surface\,tension}} = \frac{{\rho v^{2} D}}{\gamma }$$

3 $$Bo = \frac{{\rho gD^{2} }}{\gamma }$$

where *ρ* is the fluid density, *η* represents the shear viscosity, *γ* is the surface tension, *D* is the drop diameter, and *v* denotes the drop velocity. The *Re* represents the balance between inertial force and viscous force, and the Weber number represents the ratio of inertial force to capillary force. The effect of gravity is characterized by the Bond number, which can be neglected in most cases for micron-sized drops. The ratio between $Re$ and $We$^1/2^, the inverse of the Ohnesorge number, relates the viscous forces to surface tension forces. Often defined as the Z number, it is used to predict the jetting behavior of drops from Drop-on-Demand (DoD) printhead.

4 $$Z = \frac{1}{Oh} = \frac{Re}{{\sqrt {We} }} = \frac{{\sqrt {\rho \gamma D} }}{\eta }$$

It is widely assumed that Z is required to be in the 1 < Z < 14 range for proper drop formation^16,17^.

A drive waveform is a set of timed actuator wall movements that generate and propagate acoustic pressure waves within a channel. The drive waveform has a critical role in the operation of the printhead with the ink to be jetted^18^. Whereas the morphology of a jetted drop is mostly determined by the fluid properties, the incorrect waveform can still lead to unsatisfactory jetting with undesirable satellite formation or even non-jetting. A trapezoidal single-pulse waveform, comprised of the pulse rise, hold, and fall phases, is commonly used to drive the piezoelectric transducer. A widely adopted rule of thumb to optimize a waveform is to scan a range of hold times of the trapezoidal waveform with the predetermined rise and fall times and monitor drop formation^19^. The optimum is defined here as the highest drop velocity for a given voltage amplitude^20–22^. New methods have recently been proposed to design customized waveforms for satellite-free jetting. Kwon described a waveform design approach based on monitoring the motion of ink’s meniscus at the nozzle^11^. His algorithm determined the optimal dwell time by identifying the meniscus location using a charge-coupled device (CCD) camera and strobe LEDs. Xiao et al. designed a unipolar trapezoidal waveform through a combination of laser Doppler vibration testing and numerical simulation^23^. They first determined rise and fall times by the step response time of the piezoelectric actuated vibration plate and then optimized a dwell time by simulating the velocity wave induced by a rising or falling voltage pulse. The design of a multipulse waveform has recently been presented as a means of reducing the length of ligament or satellite drops^24^. Hur’s team utilized the lumped element model of a recirculating piezoelectric inkjet printhead to improve bipolar waveforms, demonstrating that a bipolar waveform with a second pulse voltage of 1/3 than that of the first could suppress residual vibrations within the printhead channel^25^. Despite the development in waveform design methodologies, the knowledge of wave propagation and drop formation is limited, and waveform optimization still requires time-consuming and repetitive waveform parameter modifications, while monitoring obtained jetting images. In addition, the previous experimental studies on the design of an optimal waveform were conducted with one or more limited number of inks for a given printhead^26^, but it is difficult to apply the proposed waveforms to other ink systems with diverse fluid characteristics.

Meanwhile, smart manufacturing technology has incorporated artificial intelligence (AI) into many different areas of the production process, such as optimization, control, and troubleshooting^27–29^. Machine learning methods are now being applied in the inkjet printing process. Wu and Xu utilized ensemble learning techniques to design data-driven models to predict drop velocity and volume^30^. Ogunsany et al. presented a vision-based approach for in-situ monitoring of drop formation^31^. They classified the state of the jetting behavior from analyzed captured image data using a back-propagation artificial neural network method. Huang and his colleagues proposed an unsupervised learning framework for video data of jetting process to better understand the drop flow pattern and its underlying mechanism^32^. They demonstrated that a deep recurrent neural network could learn the drop motion at the nozzle and anticipate drop evolution behavior. However, no framework has been devised by any AI approach to recommend drive waveforms in high-quality inkjet printing of a wide range of printable inks.

This study proposes a closed-loop machine learning algorithm for designing an optimal drive waveform for reliable satellite-free inkjet printing. Representative 11 model inks whose Z numbers are between 1 and 50 were ink-jetted to collect the drop formation and velocity dataset. For each ink, 1100 distinct designs of waveforms were applied to a printhead, and its jetting images were obtained using a high-speed imaging technique. Five machine learning models were utilized to predict the characteristics of the jetting behavior, and their performances were compared to determine the most suitable. Following the development of a predicted model, we established a closed-loop algorithm for recognizing the optimal set of waveform parameters for a satellite-free drop formation. Finally, our proposed method was validated by printing an unknown ink with a recommended waveform at a target velocity.

## Materials and methods

### Inkjet printing system and process

A home-built inkjet apparatus was employed for this experiment. The system comprised a piezoelectric inkjet nozzle with a diameter of 40 μm (MJ-ATP-01-040 DLC, MicroFab Technologies), a waveform driver (JetDrive, MicroFab Technologies), a nano-pulsed flashlight source (NP-1A, SUGAWARA Laboratories Inc), a CCD camera (avA1000-100gm, Basler) with a high magnification zoom lens (Zoom 6000 Lens System, Navitar), and the 5 × objective lenses (Infinity Corrected Long Working Distance Objective, Mitutoyo). The inkjet nozzle belongs to a squeeze-mode design where a piezo transducer wrapped the outside of a glass capillary tube. The inkjet nozzle pushes out the liquid when receiving the trigger pulse at a jetting frequency of 10 Hz. Based on single-flash high-speed imaging, all jetting images were obtained by the ultra-short pulse flashlight and the CCD camera. The drop watcher system employs a stroboscopic principle to capture the jetting image. The strobe trigger delay time can be adjusted in 1 μs increments. The very short flashlight with a duration of 180 ns avoided significant motion blurring. These components were electrically synchronized and controlled by an embedded controller (cRIO-9035 and NI-9401, National Instruments).

### Model inks

Deionized water, ethylene glycol, glycerol, and their different mixtures were used as model inks. Information on the fluid properties of those inks is presented in Table 1. Their shear viscosities were measured using a cone-plate viscometer (DV2T, Brookfield) at shear rates of 100–1000 s^−1^. All inks were Newtonian fluids since the viscosity remained constant in the measured range. Equilibrium surface tension was evaluated with a bubble pressure tensiometer (Proline t15, SITA Lab Solution). Each ink sample was filtered with a 0.45 μm hydrophilic nylon membrane before jetting.

**Table 1:** Summary of fluid properties for each ink, together with dimensionless number Z.

Ink No	Composition (volume fraction)			Density (kg m^−3^)	Viscosity (mPa s)	Surface tension (mNm^−1^)	Z	Training
	Deionized water	Ethylene glycol	Glycerol					
1	100	0	0	998.4	1.000	71.6	53.6	Used
2	90	0	10	1037.0	1.613	69.3	33.2	Used
3	80	0	20	1066.5	1.843	68.4	29.3	Used
4	70	0	30	1093.2	2.773	67.3	19.5	Used
5	60	0	40	1126.3	4.140	66.5	13.2	Used
6	90	10	0	1036.7	1.223	67.0	43.1	Used
7	70	30	0	1043.3	1.990	61.3	25.4	Used
8	40	60	0	1046.7	4.520	52.3	10.5	Used
9	30	70	0	1094.4	5.940	50.1	7.88	Used
10	20	80	0	1106.8	8.883	49.3	5.26	Used
11	0	100	0	1113.5	16.38	45.3	2.74	Used
12	50	50	0	1071.1	3.450	56.4	14.3	Unused

### Jetting data collection

Each ink was printed with 1100 different shapes of drive waveforms whose parameters such as voltage, rise, dwell and fall time were randomly selected in the range in Table 2, and their jet formation data were produced. The waveform is a trapezoidal pulse comprising driving voltage as well as rise, dwell, and fall times. A LabVIEW™ program was written to repeat the waveform setting–jetting–imaging sequence with increasing flash delay times to capture a series of drop formation images automatically. The delay times were adjusted from 40 to 400 μs with a 5 μs interval. A total of 883,000 jetting images were acquired and stored for the application of the machine learning models. The NI Vision Development Module was used to improve image quality, identify jets and drops, and extract quantitative information, including the number of the produced drops and their velocities. The drops were obtained by counting the main and satellite drops during travelling. The positions of the main drop at different trigger times were recorded and used to calculate the drop velocity.

**Table 2:** Ranges of each parameter in waveform.

Parameters of waveform	Low level	High level
Voltage (V)	10	80
Rise time (μs)	2	40
Dwell time (μs)	2	60
Fall time (μs)	2	40

### Machine learning models

Five machine learning algorithms, such as Support Vector Machine (SVM)^33^, k-Nearest Neighbor (kNN)^34^, Random Forests (RFs)^35^, Extreme Gradient Boosting (XGBoost)^36^, and Multi-layer Perceptron (MLP)^37^, were implemented to predict drop velocity and formation in this work. All models were programmed using the python scikit-learn package with XGBoost library. We employed the receiver operating characteristic (ROC) curve^38^ and precision-recall (PR) curve^39^ to evaluate the predictive ability of the learning models. When evaluating the performance of a machine learning model, the ROC curve demonstrates diagnostic ability, and the PR curve summarizes the trade-off between the true positive rate and the positive predictive value for the learning model from diverse probability thresholds for classification.

## Results

### Overall workflow of the machine learning

**Figure 1:**
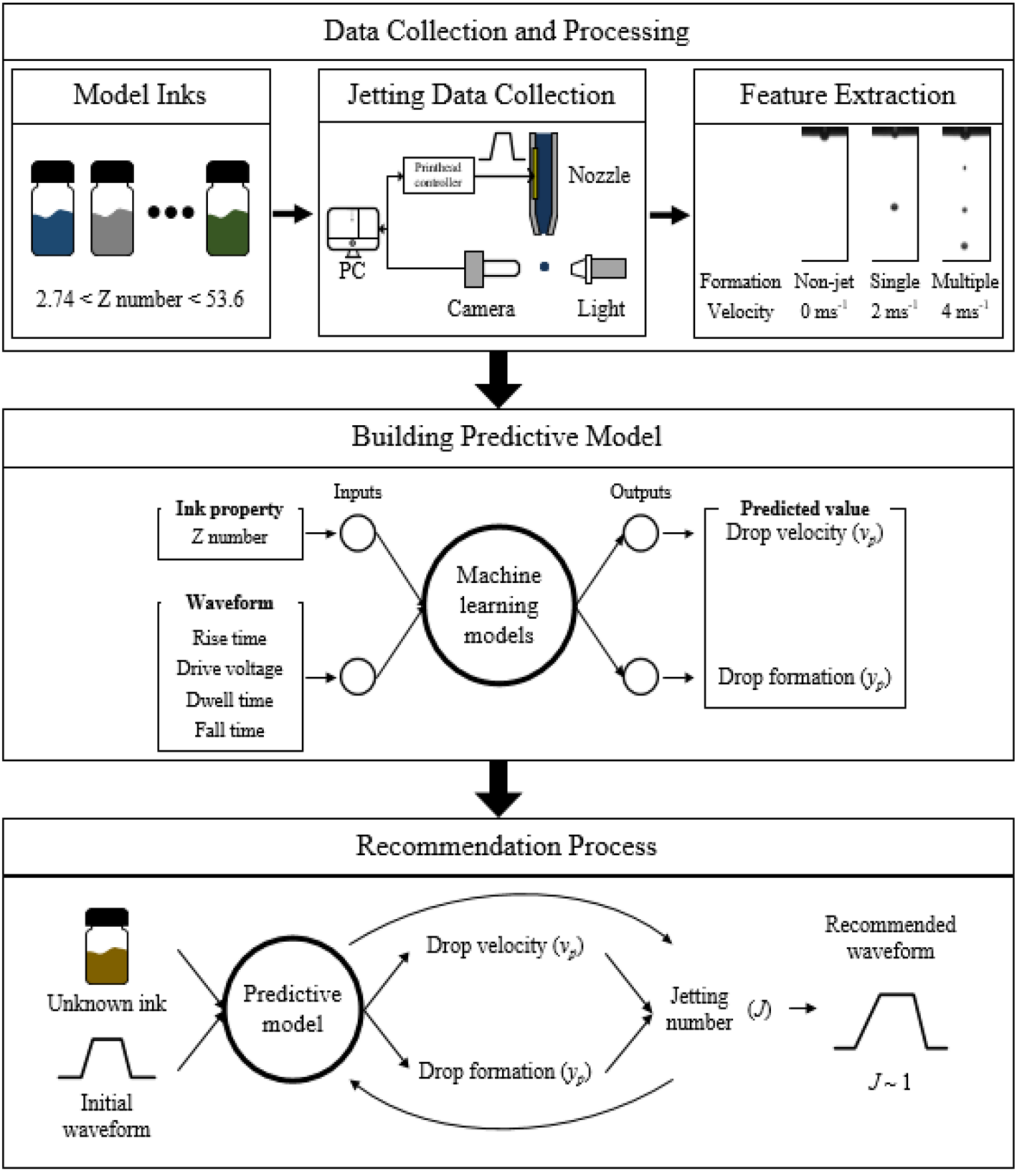
Schematic representation of the working flow for designing an optimized inkjet drive waveform using machine learning models.

Figure 1 shows the machine learning process for the recommendation of a waveform for satellites-free jetting behavior. The process is comprised of collecting jetting images, building a predictive model, and finding an optimal waveform for reliable single drop jetting. Initially, we collected high-speed jetting images on various fluid properties of inks with different shapes of actuation waveforms, which were employed to extract jetting morphologies and velocity. We categorized jetting morphologies into three regimes of non-jetting (designated as class 0), satellite-free single drop formation (class 1), and multiple drop formation (class 2). The drop formation of jetting behavior and velocity of jets under given conditions were examined from the obtained high-speed images. We then employed five distinct machine learning models, such as Support Vector Machine (SVM), k-Nearest Neighbor (kNN), Random Forests (RFs), Multi-layer Perceptron (MLP), and Extreme Gradient Boosting (XGBoost), and compared their performances to determine the outstanding model for this study. After constructing a predictive model, we established a closed-loop algorithm, which identifies an optimal waveform based on the predictive model. We confirmed that the recommendation stage successfully obtained the optimal waveform of an unknown ink by using the predictive model. Finally, we verified the model using an unknown ink.

### Jetting data collection for various inks and waveforms

To collect jetting data and determine how modifying fluid properties of ink and a design of a drive waveform affected jetting behavior, we inkjet-printed 11 different model inks with a different Z number ranging from 2 to 53 by blending deionized water, ethylene glycol, and glycerol. Each ink was ejected with our home-built inkjet system integrated with a high-speed imaging apparatus considering rapid single-flash photography. The rise/fall, dwell time and peak drive voltage of the analog trapezoidal driving pulse were adjusted between 2 and 40 μs, 2 and 60 μs, and 10 and 80 V, respectively. Consequently, each ink was ejected with a total of 1100 distinctly designed jetting waveforms, and their jetting behaviors were recorded with 73 high-speed images for each waveform. The images were used to extract jetting regime and velocity for machine learning.

**Figure 2:**
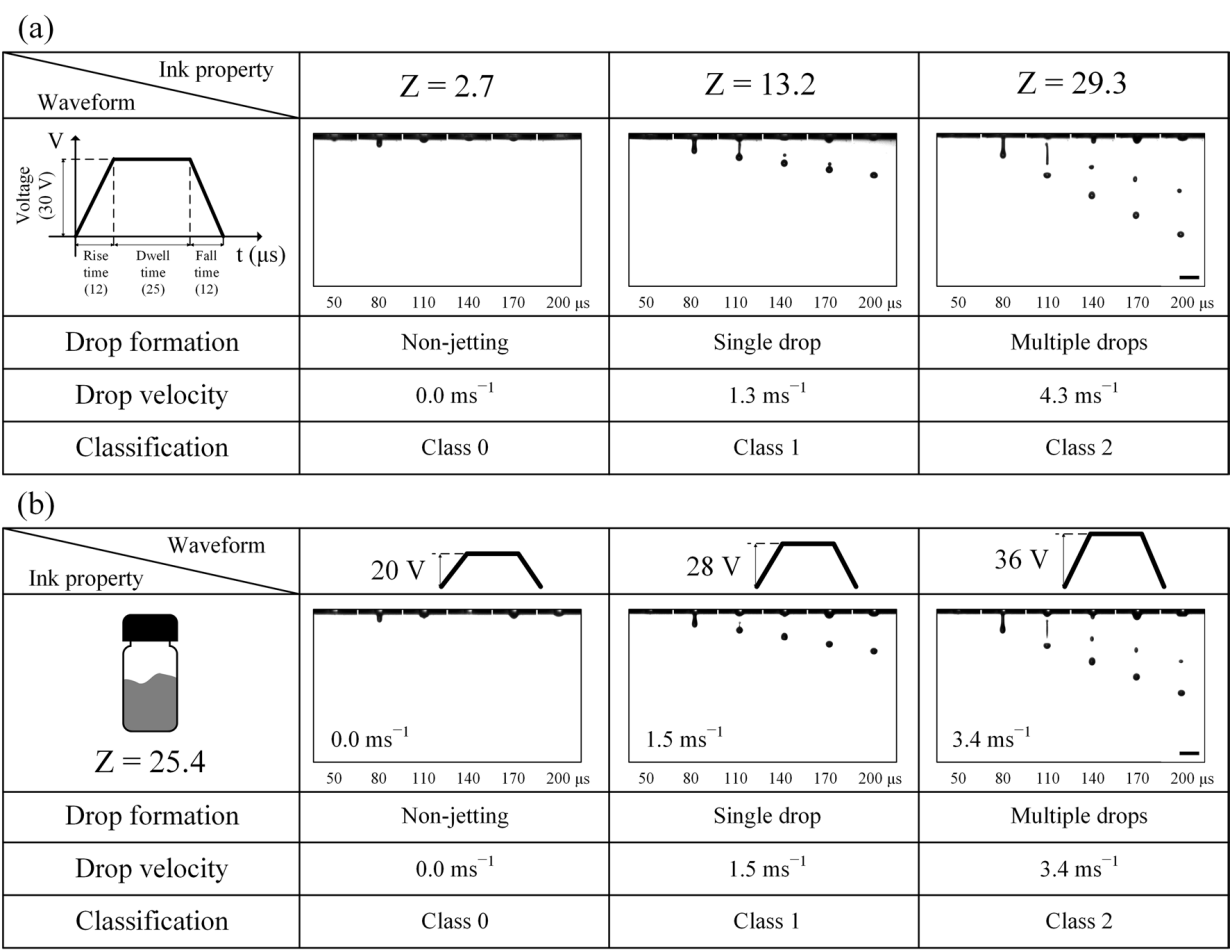
The influence of waveform design and ink property on drop formation and drop velocity. (**a**) Ink property changes with a waveform unchanged. (**b**) Amplitude of a waveform increased for a model ink. Scale bar: 100 μm.

Images of jetting morphologies depending on Z numbers and waveform design are shown in Fig. 2. The increase in Z numbers resulted in various jetting morphologies. For example, Fig. 2a indicates that no drops were generated at a Z number of 2.7, and the single drop formation was achieved by increasing the Z number to 13.2. Further alteration in fluid properties for a higher Z number of 29.3 led to the formation of the main drop accompanied by an unwanted satellite. The waveform remained the same during the experiments. We then examined the influence of the waveform design on jetting behavior for a given ink with a Z value of 25.4. As seen in Fig. 2b, 20 V amplitude of a trapezoidal waveform did not drive an ink drop out of the nozzle; however, an increase to 28 V with rise/fall and dwell times unchanged led to a desirable single main drop. However, satellites were formed for further increased drive voltage.

### Predictive model by machine learning

After the collected vision data and their analyses were established, we first identified a total of 5 inputs that influence drop formation and velocity. The drop formation was categorized into three classes based on the number of drops. Class “0” indicates no drop formation, class “1” single drop formation, and class “2” satellites formation. The inputs indicate the ink properties represented by the Z number and the waveform consisted of voltage amplitude, rise, dwell, and fall times. A set of five supervised learning models, including SVM, kNN, RFs, MLP, and XGBoost, were trained by the datasets. To validate the models and decrease the risk of overfitting, the dataset was divided into a training set (60%), a validation set (20%), and a test set (20%).

**Figure 3:**
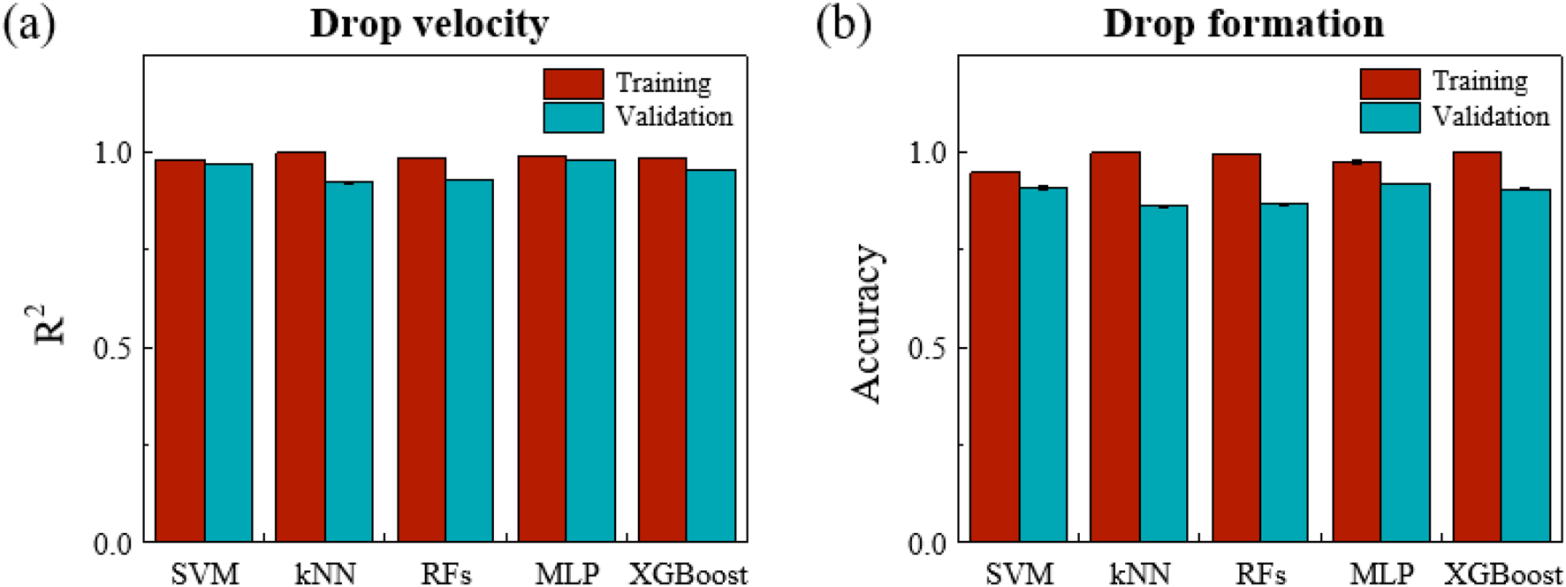
Performance evaluation of 5 machine learning models in predicting drop velocity and drop formation. (**a**) coefficient of determination of predicting drop velocity (**b**) accuracy of predicting drop formation. SVM, Support Vector Machine; kNN, k-nearest neighbors; RFs, random forests; MLP, Multi-layer Perceptron; XGBoost, extreme gradient boosting.

The hyper-parameters of the learning model are summarized in Table 3; the values are tunned based on the performance on the validation set. We evaluated their performance in terms of drop velocity and drop formation. The coefficient of determination (R^2^) for regression models was examined to predict drop velocity. Fig. 3a illustrates that all of the five machine learning models can be predicted with a high R^2^ of > 90%. The overall classification performances of SVM, kNN, RFs, MLP, and XGBoost were evaluated by the accuracy as displayed in Fig. 3b. The accuracy values were 0.92, 0.91, 0.86, 0.91, and 0.87 for MLP, SVM, kNN, XGBoost, and RFs, respectively. Among these models, the MLP, SVM, and XGBoost models outperform kNN and RFs, with high prediction accuracy and no significant overfitting in both outcomes.

**Table 3:** Summary of important hyper-parameters of machine learning models.

Learning model	Hyper-parameters	
	Regression	Classification
SVM	C = 10, gamma = 0.3	C = 10, gamma = 0.5
kNN	k = 10	k = 20
RFs	Max_depth = 20	Max_depth = 20
MLP	1st layer size = 200 2nd layer size = 100	1st layer size = 100 2nd layer size = 100
XGBoost	Max_depth = 5	Subsample = 0.7 Min_child_weight = 20

**Figure 4:**
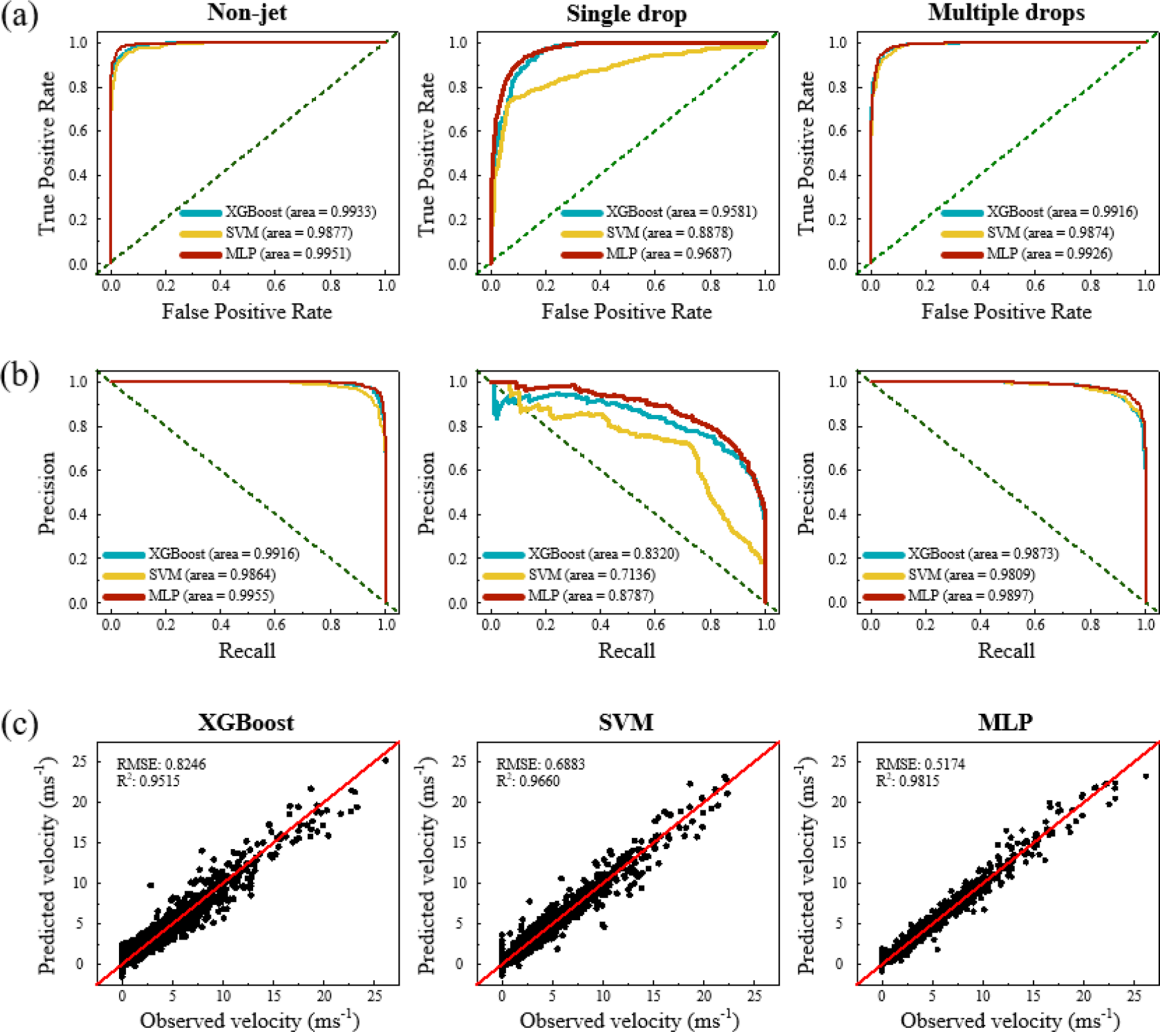
(**a**) Receiver operating characteristic curve and (**b**) precision-recall curve of predicting drop formation applying the test set in SVM, XGBoost, and MLP models. (**c**) Comparison of observed and predicted drop velocity using XGBoost, SVM, and MLP in the test set. The green dash line indicates the randomly guessing baseline.

Three preferable models, such as SVM, XGBoost, and the MLP were further compared in detail. During the classification challenge, we constructed receiver operating characteristic (ROC) curves to evaluate the prediction performance of the models. The ROC curve displays the trade-off between the true positive rate and the false positive rate as shown in Fig. 4a. It took the uncertainty of each prediction into account. The deviation of the curve toward the upper left corner from the random guess baseline that is displayed to the green dash line in Fig. 4a indicated higher prediction accuracy. MLP demonstrates the highest deviated ROC curve compared with those of SVM and XGBoost of each class. MLP also reveals the highest value of the area under the ROC curve (AUC), which is equivalent to the probability that the classifier will rank as a randomly chosen positive instance higher than a randomly chosen negative instance in comparison with SVM and XGBoost. The AUC value of MLP was particularly significantly higher than other learning models in class “1.” The overall classification performance of MLP, SVM, and XGBoost was evaluated through the precision-recall (PR) curves as an additional indicator. In contrast to the ROC curve, the more the deviation of a PR curve toward the top right corner from the randomly guessing baseline indicates that a machine learning model achieves higher prediction accuracy. MLP also delivered high predictive performance, as presented in Fig. 4b. MLP obtained a precision of 0.84 at the recall of 0.73 in class “1,” which is substantially higher than those of SVM and XGBoost. In the regression problem, most algorithms showed useful agreement between the observed and predicted velocities as shown in Fig. 4c. MLP also provided the highest R^2^ of 0.9815 and the lowest root mean square error of 0.5174. We discovered a strong predictive model, which suggests that the MLP model would be most suitable for predicting drop velocities and drop formation.

### A closed-loop AI algorithm for identifying the optimal waveform

**Figure 5:**
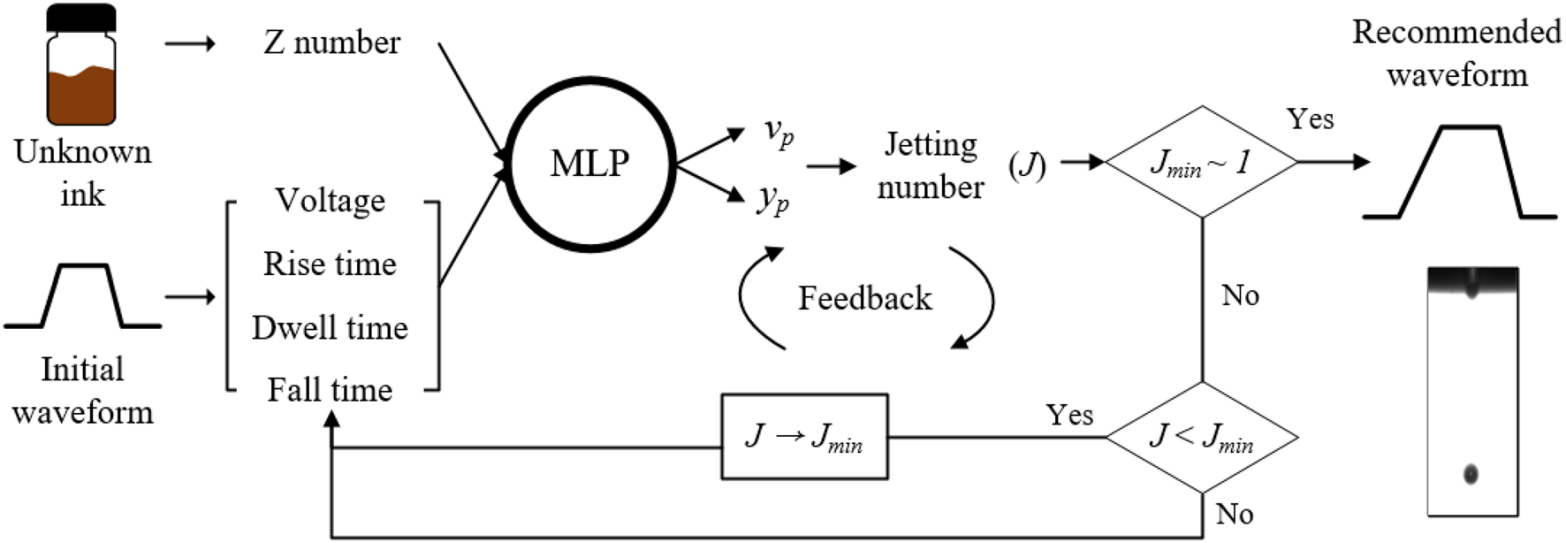
Schematic diagram of a waveform recommendation process.

Having evaluated the performances of representative machine learning models, we chose the MLP model to build an optimization algorithm for identifying the optimal waveform. The MLP model deployed initially cannot provide any opportunity for improvement. Therefore, we have built a closed-loop AI algorithm, the waveform design generated by which can be continuously optimized as machine learning improves with data and experience. The algorithm corresponding to the closed-loop waveform optimization is outlined in Fig. 5. The algorithm is performed in two phases. In the first phase, the input layer of the MLP receives the input data for an ink property (Z number) and an initial drive waveform (voltage, rise/dwell/fall times) to be processed. The required task on the prediction of drop velocity (*v*_p_) and drop formation (*y*_p_) is performed by its output layer. A double hidden layer that is placed in between the input and output layer is the actual computational engine of the model. The second phase is used to actively update the MLP by taking feedback from the outputs.

To form a closed-loop for determining an optimal waveform, we define the jetting number (*J*) as follow:

5 $$J\left( {v_{p} ,v_{t} ,y_{p} } \right) = \underbrace {{\left( {v_{p} - v_{t} } \right)^{2} }}_{error\,of\,drop\,velocity} + \underbrace {{\left\{ {y_{p} \left( {2 - y_{p} } \right) + \delta } \right\}^{ - 1} }}_{error\,of\,drop\,formation}$$

where *v*_*p*_ and *v*_*t*_ are MLP-predicted and target drop velocity, respectively, and *y*_*p*_ represents the class of drop formation predicted by MLP, and *δ* denotes a minimal value to avoid divergence. The jetting number consists of the sum of terms reducing the error of drop velocity and formation. The former term is the squared prediction error for target drop velocity, which encourages a predicted drop velocity to reach target velocity. The latter term aims to search for a class of single drop formation. The algorithm iterates until the calculated value of *J* is close to 1, recommending an optimal set of waveform parameters for satellite-free drop formation at a target velocity.

### Verification

**Figure 6:**
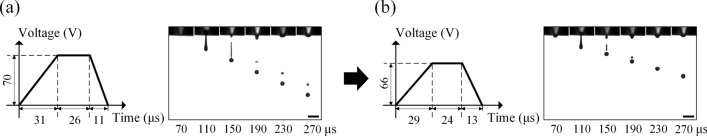
(**a**) Sequence images of drop formation at an initial waveform and (**b**) an optimized waveform from the proposed method. Scare bar: 100 μm.

**Figure 7:**
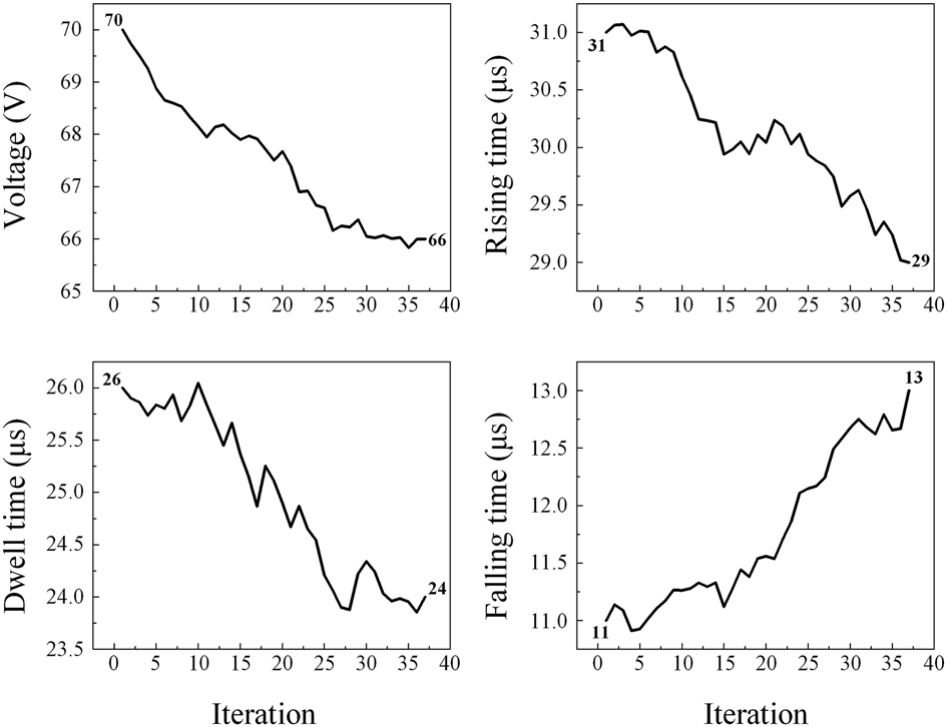
Change of waveform parameters for iteration.

To verify the proposed method, we prepared an ink with Z = 14.25 that had not been used for training. The target velocity was set to 1.5 ms^−1^ to calculate the jetting number. In Fig. 6a, the double drops were ejected at the initial waveform (peak voltage = 70 V, rising time = 31 μs, dwell time = 26 μs and falling time = 11 μs). The velocity of the main drop was 2.54 ms^−1^. After using the proposed method, the waveform factors were adjusted for voltage, rise/dwell/fall times as 66 V, 29 μs, 24 μs, and 13 μs, respectively. In Fig. 6b, double drops were formed, yet the second drop merged with the main drop at 1.62 ms^−1^ after an elapsed time of 220 μs. The fall time was increased by 2 μs and the voltage, rise time, and dwell time were reduced by 4 V, 2 μs, and 2 μs, respectively. The parameters of the waveform change according to iteration as displayed in Fig. 7. The change of the waveform occurs, causing the drop velocity to decrease. These findings suggest that MLP recognizes the need to reduce voltage and pulse width to reduce drop velocity.

## Conclusion

In this study, we introduced a closed-loop machine learning process to recommend the optimal design of a drive waveform for satellite-free inkjet printing. We created and collected the big datasets of drop formation and velocity with 11 representative inks with different fluid properties and 1100 distinct waveform designs. We concluded that MLP achieved the best prediction of drop formation and drop velocity among the five representative machine learning models tested. We developed the closed-loop algorithm to recommend the optimal waveform with the Jetting number. The model was verified with an unknown ink for target drop velocity of 1.5 ms^−1^ and single drop formation. We anticipate that our approach can be applied to a wide range of Newtonian inks and industrial printheads. Future work will establish an advanced machine learning process with non-Newtonian inks and bipolar waveform designs.
